# Super-Low Dose Lipopolysaccharide Dysregulates Neutrophil Migratory Decision-Making

**DOI:** 10.3389/fimmu.2019.00359

**Published:** 2019-03-12

**Authors:** Brittany P. Boribong, Mark J. Lenzi, Liwu Li, Caroline N. Jones

**Affiliations:** ^1^Genetics, Bioinformatics, and Computational Biology, Virginia Polytechnic Institute and State University, Blacksburg, VA, United States; ^2^Department of Biological Sciences, Virginia Polytechnic Institute and State University, Blacksburg, VA, United States

**Keywords:** neutrophils, chemotaxis, gradients, sepsis, neutrophil memory, lipopolysaccharide, microfluidics

## Abstract

Neutrophils are the first responders to infection and play a pivotal role in many inflammatory diseases, including sepsis. Recent studies have shown that lipopolysaccharide (LPS), a classical pattern recognition molecule, dynamically programs innate immune responses. In this study, we show that pre-treatment with super-low levels of LPS [1 ng/mL] significantly dysregulate neutrophil migratory phenotypes, including spontaneous migration and altering neutrophil decision-making. To quantify neutrophil migratory decision-making with single-cell resolution, we developed a novel microfluidic competitive chemotaxis-chip (μC^3^) that exposes cells in a central channel to competing chemoattractant gradients. In this reductionist approach, we use two chemoattractants: a pro-resolution (N-Formyl-Met-Leu-Phe, fMLP) and pro-inflammatory (Leukotriene B_4_, LTB_4_) chemoattractant to model how a neutrophil makes a decision to move toward an end target chemoattractant (e.g., bacterial infection) vs. an intermediary chemoattractant (e.g., inflammatory signal). We demonstrate that naïve neutrophils migrate toward the primary end target signal in higher percentages than toward the secondary intermediary signal. As expected, we found that training with high dose LPS [100 ng/mL] influences a higher percentage of neutrophils to migrate toward the end target signal, while reducing the percentage of neutrophils that migrate toward the intermediary signal. Surprisingly, super-low dose LPS [1 ng/mL] significantly changes the ratios of migrating cells and an increased percentage of cells migrate toward the intermediary signal. Significantly, there was also an increase in the numbers of spontaneously migrating neutrophils after treatment with super-low dose LPS. These results shed light onto the directional migratory decision-making of neutrophils exposed to inflammatory training signals. Understanding these mechanisms may lead to the development of pro-resolution therapies that correct the neutrophil compass and reduce off-target organ damage.

## Introduction

Recent studies suggest that neutrophils are a key player in the development of sepsis, the current leading cause of death in hospitals (1–4). Neutrophils are the most abundant white blood cells (~60%) and are the first responders to infection and inflammation. Neutrophils can navigate effectively through complex tissue microenvironments toward pathogens and play a critical role in controlling infection under normal conditions (5). By necessity, in the setting of multiple chemoattractants, neutrophils must prioritize, favoring end target chemoattractants (e.g., N-Formyl-Met-Leu-Phe, fMLP) emanating from the site of infection over intermediary endogenous chemoattractants [e.g., Leukotriene B_4_ (LTB_4_) and interleukin-8 (IL-8)] encountered en route to sites of infection (6). In septic patients, neutrophils migrate and accumulate in healthy organs instead of migrating toward the infection (2). However, lack of control of the tissue microenvironment and the complexity of tracking the trajectories of immune cells *in vivo* prohibits the study of cell migratory decision-making.

**Figure 1:**
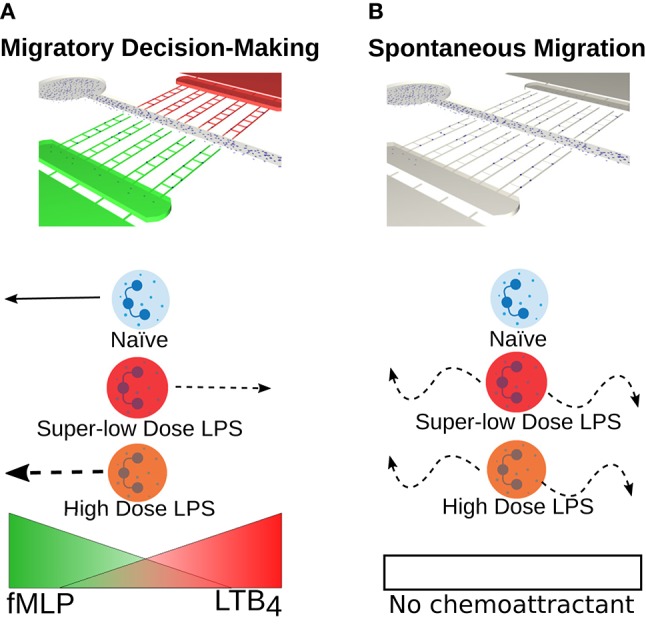
Super-low dose LPS primes neutrophils for dysfunctional migratory decision-making and increases spontaneous migration. **(A)** Schematic illustrating a novel microfluidic competitive chemotaxis-chip (μC^3^) that generates a competitive chemoattractant environment and enables the quantification of neutrophil migratory decision-making. Healthy neutrophils (blue) are known to preferentially migrate toward a primary, end target chemoattractant (fMLP) over a secondary or intermediary chemoattractant (LTB_4_) (6). However, for the first time we show that migratory decision-making process is affected by priming and tolerance induced by LPS stimulation of varying orders of magnitude. In this study, we demonstrate a shift in chemoattractant preference toward an inflammatory signal are primed with a super-low dose of LPS [1 ng/mL] (red neutrophil) and an increase in magnitude of the migratory response when neutrophils are primed with a high dose of LPS [100 ng/mL] (orange neutrophil). **(B)** Schematic illustrating neutrophil migration in the absence of chemoattractant with and without LPS stimulation. Healthy neutrophils (blue) do not migrate in the absence of a chemoattractant. In this study, we show that super-low (red) and high (orange) levels of LPS stimulation both induce spontaneous neutrophil migration in the absence of a chemoattractant.

Previous work from us described a dysfunctional migration phenotype, including spontaneous migration, in neutrophils isolated from septic burn patients (7). Emerging studies suggest that dynamic programming of neutrophils may induce distinct memory states that influences cell phenotype (8, 9). Exposure to pro-inflammatory cytokines, chemokines, mitochondrial contents, and bacterial and viral products induces neutrophils to transition from a basal state into a primed one, which is currently defined as an enhanced response to activating stimuli (10). Phenotypic changes associated with priming also include activation of a subset of functions, including chemotaxis (3, 11–13). Recent studies from our group have suggested that neutrophil “priming or memory” may play a role in the dysfunction of neutrophils during sepsis. In chronic diseases, it has been shown that super-low levels of LPS prime monocytes, and most likely neutrophils (8, 14–17), for a dysfunctional and intense response to a secondary infection. It is unknown how this neutrophil memory affects cells migration. Previous studies on the hierarchies of chemoattractants show that neutrophils favor primary signals from pathogens over secondary inflammatory signals (18). This makes sense because the primary function of the immune system is to fight infectious invaders. However, these studies only studied the behavior of naïve neutrophils and failed to address the migratory decision-making of “pre-conditioned” memory neutrophils previously exposed to microbial/inflammatory signals. A previous study that examined migration phenotypes of stimulated neutrophils, found neutrophils to favor primary pro-resolution signals over a pro-inflammatory signals (19). However, this study focused on high-dose endotoxin priming [10 ng/mL]. Our study aims to understand the changes in migration patterns caused by neutrophil pre-conditioning with both super-low and high dose of LPS. To achieve this objective, we examined two neutrophil phenotypes: (1) migratory decision-making (Figure 1A); and (2) spontaneous migration (Figure 1B) following pre-conditioning with varying dosages of LPS. We quantified how super-low dose and high dose LPS pretreatment affects these phenotypes as compared to the “healthy,” untreated naïve cells (Figure 1). We hypothesized that the neutrophil migratory decision-making may be differentially affected by varying signal-strengths of LPS pre-conditioning.

**Figure 2:**
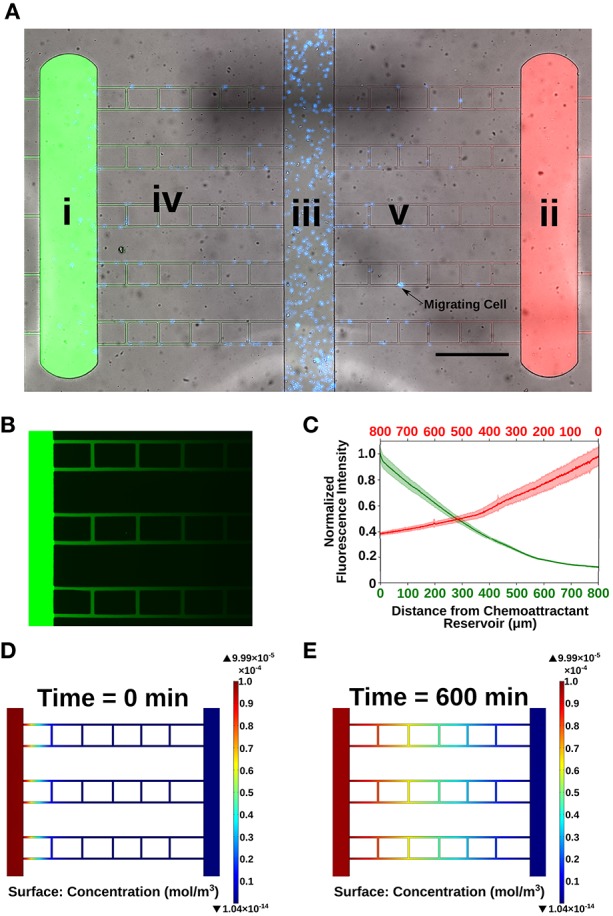
Characterization of microfluidic competitive chemotaxis-chip (μC^3^). **(A)** Microfluidic device designed to measure neutrophil migratory decision-making in a competitive chemoattractant environment. (i) Chemoattractant reservoir for end target chemoattractant (fMLP [10 nM], green). (ii) Chemoattractant reservoir for intermediary chemoattractant (LTB_4_ [100 nM], red). (iii) Central loading channel for neutrophils (blue). (iv) Migration channels for cells to migrate from the central loading channel to the chemoattractant reservoirs (10 × 10 μm). A linear gradient is formed along the length of the migration channels within 15 min. of priming chemoattractants into the reservoirs. (v) Ladder maze designed to measure directional chemotaxis of neutrophils. Chemoattractant gradient within ladder rungs 10-fold less than in straight migration channels. 10X Bright-field, FITC, TRITC-merged image taken in Nikon TiE. Scale bar = 500 μm. **(B)** Image of experimental chemoattractant gradient (green) with FITC-conjugated dextran (MW = 10,000 Da) to illustrate formation of linear gradient within the microfluidic device. **(C)** Quantification of the dual gradients formed within the microfluidic device. Slopes of gradients for fMLP (MW = 438 Da) and LTB_4_ (MW = 336 Da) are equal (within 5%) (*n* = 10 separate linear channels). **(D,E)** COMSOL finite element modeling of chemoattractant gradient over the duration of the experiment (600 min). A gradient is formed within the migration channels from the chemoattractant reservoir to the central loading channel and stays stable throughout the duration of the experiment.

To understand neutrophil function, it is important to recapitulate these complex gradients to more accurately depict *in vivo* migration responses in an *in vitro* experimental model. The gold standard to measure chemotaxis is the transwell assay or Boyden Chamber (20). This assay lacks temporal resolution and measures only end-point neutrophil accumulation, and cannot measure individual cell velocity or directionality. To address these shortcomings, researchers have developed microfluidic assays to study cell chemotaxis in spatiotemporally controlled gradients (21–24). We report a novel microfluidic competitive chemotaxis-chip (μC^3^) that enables the measurement of neutrophil migration in the presence of dual gradients (Figures 1, 2A, Table 1). The μC^3^ allows us to define the competitive migratory behavior of neutrophils with high spatial and temporal resolution. This device is easy to use and does not require valves. Our device incorporates migration channels and mazes that we have recently reported, which will allow us to measure neutrophil directionality (1, 25). Neutrophil migration in confined channels is more directional, easier to quantify and more accurately models migration within the tissue compared to standard planar migration assays (26, 27). Using this device, we examined the migratory decision-making process of dHL-60 cells, a model neutrophil cell line, in the presence of two competing chemotaxis stimulants, LTB_4_ and fMLP. fMLP is a synthetic peptide that resembles bacterial byproducts and is a very powerful chemotactic factor. LTB_4_ is a potent lipid mediator of allergic and inflammatory reactions, as well as a potent modulator of neutrophil chemotaxis (28). Furthermore, we use this system to examine the decision-making memory dynamics of dHL-60 neutrophil-like cells pre-challenged with super-low vs. high-dose of LPS (Figure 1). dHL-60 cells have been extensively characterized and are an accepted model for human peripheral blood neutrophils. Chemokinetic and chemotactic responses to chemotactic peptide are similar for dHL-60 cells and human peripheral blood neutrophils, and mean speed of migration, the fraction of migrated cells and the concentration of stimulus optimal for activation are similar (29–33). Furthermore, these cells have been utilized in previous microfluidic migration assays and work well in these platforms (26, 34, 35). Using dHL-60 cells in our studies enables us to prime cells with varying levels of LPS overnight without effecting the functionality of the cells, as would be the case with primary human peripheral blood neutrophils that have a short half-life (12 h) and lose functionality after being isolated from the blood microenvironment. Recent studies have shown that antioxidant preservatives can be used to extend the functionality of isolated human peripheral blood neutrophils and in future studies we could test LPS priming on primary human cells (36). We will also use primary neutrophils isolated from patients with varying levels of blood LPS (e.g., septic patients), however this will increase variability in samples and prohibit precise control of neutrophil priming conditions. For this proof-of-concept study, dHL-60 cells are an ideal model to allow precise control of cell priming with LPS and to reduce variability in cell decision-making in the presence of defined dual chemoattractant gradients. Our study provides novel insight toward neutrophil decision-making that may model dysregulated neutrophil behavior seen in sepsis.

**Table 1:** Migration Parameter definitions.

**Migratory phenotype**	**Definition**	**Units**
Percentage of cells migrated (chemoattractant reservoir or migration channels)	Number of Cells in the Chemoattractant Reservoir or Migration Channels at Each Time Point/Average Number of Cells in the Center Loading Channel Throughout Entire Experiment * 100	Percentage (%)
Rate of accumulation	Slope of the 1 h in which cells accumulated in the chemoattractant reservoir the greatest	% cells migrated/hour
dHL-60 Cell velocity	Distance Cell Traveled/ Time Elapsed	μm/min
Non-directional migration	dHL-60 cells that enter the cell mazes	Number of cells
Oscillatory migration	Cells that change directions in the x or y plane ≥ 3 times	Number of cells

## Results

### Design and Optimization of Microfluidic Platform to Study Neutrophil Programming Dynamics by Super-Low Dose Endotoxin

We have designed a novel microfluidic competitive chemotaxis-chip (μC^3^) that enables the formation of a dual, competitive chemoattractant gradient. Our μC^3^ device is designed with two chemoattractant reservoirs (Figure 2Ai,ii) at opposite ends and a center cell-loading channel (Figure 2Aiii). Connecting the cell-loading channel to the chemoattractant reservoirs are 10 linear migration channels on each side (Figure 2Aiv). Chemoattractant pipetted into the chemoattractant reservoirs diffuses down through the linear channels, creating a linear gradient from the cell-loading channel to the chemoattractant reservoirs (Figure 2B). Also included in the design are vertical cell mazes to test directionality of neutrophil chemotaxis (Figure 2Av). The cell mazes have a weaker chemoattractant signal than the linear channels, which enables us to classify the cells ability to follow the stronger gradient, as well as non-persistent chemotaxis. In our experiments, we primed one chemoattractant reservoir with fMLP (a model for an end target, or pro-resolution chemoattractant) and the second chemoattractant reservoir with LTB_4_ (a model for an intermediary, pro-inflammatory chemoattractant). This allowed us to probe the decision-making dynamics of individual neutrophils. To evaluate the dynamics and stability of the chemoattractant gradients developed between the chemoattractant reservoirs and the cell-loading channel, we primed reservoir I with FITC-labeled dextran and reservoir II with TRITC-labeled dextran (both molecular mass, 10,000 Da) and measured the fluorescence levels over time. Linear gradients of chemoattractant are formed along the 900-μm-long, 10 × 10 μm cross-section migration channels. The slopes of both gradients are similar (±5%) for both chemoattractants (Figure 2C). Biophysical modeling of chemoattractant diffusion in our device using the COMSOL simulation package shows that a chemoattractant gradient along the migration channel to the central cell-loading chamber are formed in <15 min for both chemoattractants and are still present at 5 hours after the start of the experiments (Figures 2D,E).

### Priming With LPS Significantly Alters Neutrophil Migratory Decision-Making

**Figure 3:**
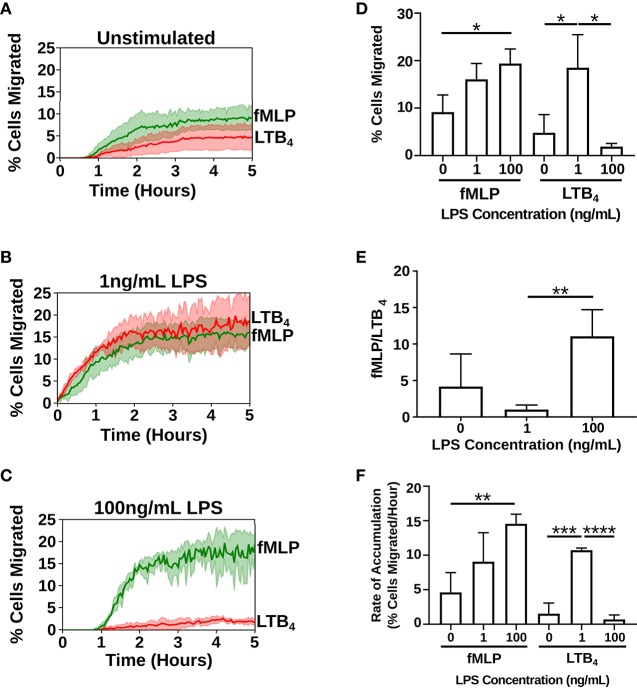
Priming with super-low dose LPS alters neutrophil migratory decision-making. Neutrophil (dHL-60 cells) migration counts in the fMLP chemoattractant reservoir (green) and LTB_4_ chemoattractant reservoir (red) over the duration of the experiment (5 h) (*n* = 3 separate experiments). **(A)** Unstimulated neutrophils preferentially migrate toward fMLP over LTB_4_. **(B)** Neutrophils stimulated with a super-low dose of LPS [1 ng/mL] preferentially migrate toward LTB_4_ over fMLP. **(C)** Neutrophils stimulated with a high dose of LPS [100 ng/mL] preferentially migrate toward fMLP over LTB_4_. **(D)** Percentage of cells migrating toward fMLP and LTB_4_ in the different treatment groups. dHL-60 cells show a significant increase in migration toward fMLP when stimulated with a high-dose of LPS. dHL-60 cells show a significant increase in migration toward LTB_4_ when stimulated with a super-low dose of LPS compared to the unstimulated cells. dHL-60 cells showed a significant decrease in migration toward LTB_4_ between the super-low dose and high-dose stimulation with LPS. **(E)** Ratio of cells migrating toward fMLP over LTB_4_. Neutrophils significantly migrate toward fMLP over LTB_4_ when stimulated with a high-dose of LPS vs. a super-low dose of LPS. **(F)** Calculated slope from the hour in which there was the highest accumulation of dHL-60 cells in the chemoattractant reservoirs. Data expressed as means and standard deviations. *p < 0.5, **p < 0.005, ***p < 0.0005, ****p < 0.0001.

We measured neutrophil chemotaxis in three different priming scenarios: unstimulated (Supplementary Video 1), stimulated with a super-low dose of LPS [1 ng/mL] overnight (Supplementary Video 2), stimulated with a high dose of LPS [100 ng/mL] overnight (Supplementary Video 3) (37). Cell counts were measured by automated counting of cells that fully migrated to the chemoattractant reservoir. Treatment with super-low dose (2% increase in cell viability compared to untreated control cells) and high dose LPS (no change compared to untreated control cells) had a negligible impact on cell viability (Supplementary Figure 2). In the unstimulated cells (Figure 3A) and the cells primed with a high dose of LPS (Figure 3C), a higher percentage of cells migrated toward fMLP over LTB_4_. Treatment with a high-dose of LPS significantly amplified the percentage of dHL-60 cells migrating toward fMLP by ~2-fold (19.4 ± 3.07. vs. 9.157 ± 3.599%) (*p* = 0.0199), while simultaneously decreasing neutrophil migration to LTB_4_ by 2-fold (1.894 ± 0.6725 vs. 4.811 ± 3.822%) (Figure 3D). Importantly, super-low dose LPS significantly amplified the percentage of cells migrating toward LTB_4_ by an order of magnitude (18.52 ± 6.944 vs. 4.811 ± 3.822%) (*p* = 0.0401) and fMLP by ~2-fold (16.06 ± 3.349. vs. 9.157 ± 3.599%) (Figures 3B,D). Significantly, programming dHL-60 cells with super-low dose LPS switched cell-decision making priority from an end target chemoattractant (fMLP) to an intermediary chemoattractant (LTB_4_). The ratio of cells prioritizing migration toward a pro-resolution signal was altered in an opposite manner with high (10:1) vs. super-low dose (1:1) LPS-programming compared to untreated control (2:1) (Figure 3E). Additionally, stimulation with LPS affected the rate of accumulation of the dHL-60 cells into the chemoattractant reservoirs (Figure 3F). Rate of accumulation was measured by calculating the slope of the hour in which there was the highest accumulation of dHL-60 cells. Stimulation with a high-dose of LPS saw a significant increase in the rate of accumulation toward fMLP in comparison to the unstimulated dHL-60 cells (14.56 ± 1.388% per hour vs. 4.604 ± 2.869% per hour) (*p* = 0.0057) (Figure 3F). Stimulation with a super-low dose of LPS also saw a ~2-fold increase in the rate of accumulation toward fMLP in comparison to the unstimulated dHL-60 cells (9.056 ± 4.209% per hour vs. 4.604 ± 2.869% per hour) (Figure 3F). Importantly, stimulation with super-low dose LPS significantly increases the rate of accumulation of dHL-60 cells toward LTB_4_ in comparison to unstimulated dHL-60 cells (10.73 ± 0.3047% per hour vs. 1.525 ± 1.552% per hours) (*p* = 0.0005) (Figure 3F). Stimulation with high-dose LPS sees a significant decrease in the rate of accumulation in comparison to the super-low dose LPS (0.6950 ± 0.6533% per hour vs. 10.73 ± 0.3047% per hour) (*p* < 0.0001) (Figure 3F).

### Single-Cell Analysis of Neutrophil Migratory Decision-Making

**Figure 4:**
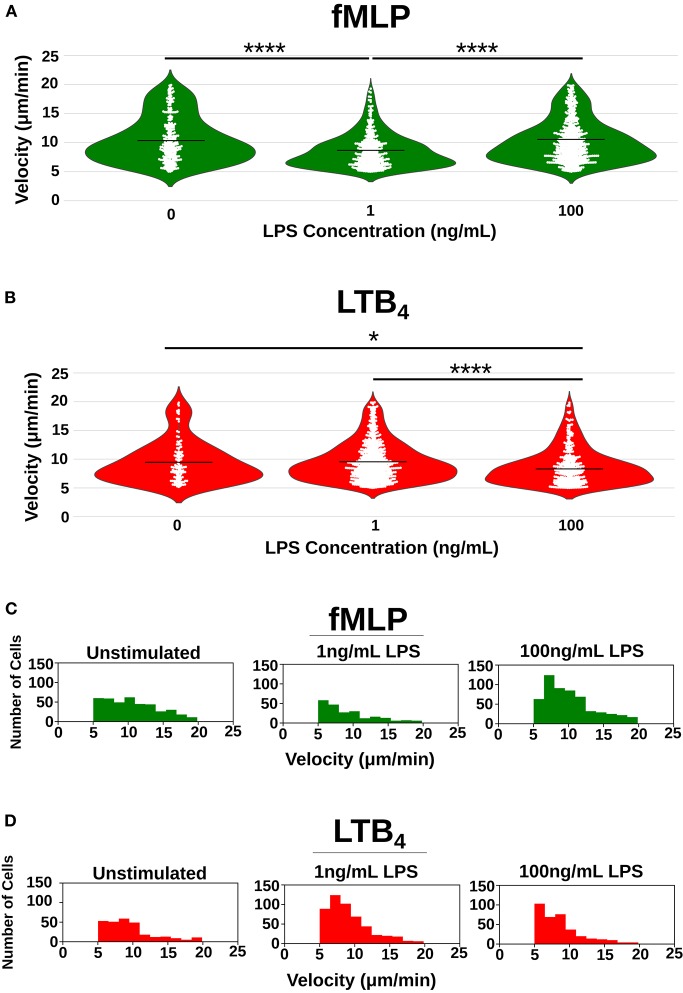
Single-cell quantification of the effect of LPS priming on neutrophil velocity. Velocity [μm/min] of individual neutrophils as they migrate toward fMLP (green) and LTB_4_ (red) recorded as a single circle on the plot. **(A)** Violin plots depicting distribution of single-cell velocities of neutrophils migrating toward fMLP in the unstimulated group (*n* = 404), super-low dose stimulated group (*n* = 222), and high-dose stimulated group (*n* = 557) where each white dot represents the velocity of a single-cell. High dose LPS [100 ng/mL] increases the mean velocity of dHL-60 cells migrating toward fMLP, whereas low dose LPS decreases the mean velocity of dHL-60 cells migrating toward fMLP. **(B)** Violin plots depicting distribution of single-cell velocities of neutrophils migrating toward LTB_4_ in the unstimulated group (*n* = 123), super-low dose stimulated group (*n* = 667), and high-dose stimulated group (*n* = 349) where each white dot represents the velocity of a single-cell. Super-low dose LPS [1 ng/mL] increased the mean velocity of dHL-60 cells migrating toward LTB_4_. **(C)** Histogram depicting distribution of velocities of neutrophils migrating toward fMLP in all three-treatment groups. Super-low dose LPS negatively shifts distribution of cell velocity, whereas high-dose LPS positively shifts distribution of cell velocities toward fMLP. **(D)** Histogram depicting distribution of velocities of neutrophils migrating toward LTB_4_ in all three treatment groups. Super-low dose LPS positively shifts distribution of cell velocity, whereas high-dose LPS negatively shifts distribution of cell velocities toward fMLP. Data is representative of one experiment, however experiment was repeated at least 3 times. *p < 0.05, ****p < 0.0001.

To further probe the differences in neutrophil migratory phenotypes caused by programming dHL-60 cells with high and super-low dose LPS, we measure the velocity of individual dHL-60 cells migrating toward both fMLP and LTB_4_ chemoattractant gradients (Table 2). Interestingly, velocity of neutrophils migrating toward fMLP significantly decreased when neutrophils were stimulated with a super-low dose of LPS (Figure 4A) but increased toward LTB_4_ (Figure 4B). The average dHL-60 velocity toward fMLP when stimulated with super-low dose LPS was 8.54 μm/min compared to 10.51 μm/min in the untreated control (*p* < 0.0001) (Figure 4A). Toward LTB_4_ the opposite effect was observed, where stimulation with super-low dose LPS resulted in an average velocity of 9.58 μm/min compared to 9.45 μm/min in the untreated control (Figure 4A). High dose LPS significantly increased migratory velocity toward fMLP compared to the super-low LPS stimulated dHL-60 cells (10.23 vs. 8.54 μm/min) (*p* < 0.0001) and significantly decreased velocity toward LTB_4_ in comparison to the super-low dose LPS stimulated cells (8.66 vs. 9.58 μm/min) (*p* < 0.0001) (Figure 4B). To better visualize the shifts in velocity caused by LPS programing, we binned velocities and graphed the number of cells migrating to each velocity range (Figures 4C,D). This clearly illustrates the shift in mid-range and high velocity dHL-60 cells toward fMLP after high dose LPS treatment compared to a shift in mid-range (Figure 4C) and high velocity dHL-60s toward LTB_4_ after super-low dose LPS treatment (Figure 4D). Quantifying single-cell phenotypes will likely be important in the future when measuring primary patient neutrophils where there may be differentially primed sub-populations of neutrophils.

**Table 2:** Migration variable summary/statistics.

	**fMLP (μm/min)**	**LTB_**4**_ (μm/min)**
Unstimulated	10.51 ± 3.91	9.45 ± 3.61
1 ng/mL LPS	8.54 ± 2.87	9.58 ± 3.36
100 ng/mL LPS	10.23 ± 3.57	8.66 ± 3.14

### Super-Low Dose LPS Treatment Increases dHL-60 Cell Oscillatory Migration Patterns and Decreases Cell Directionality

**Figure 5:**
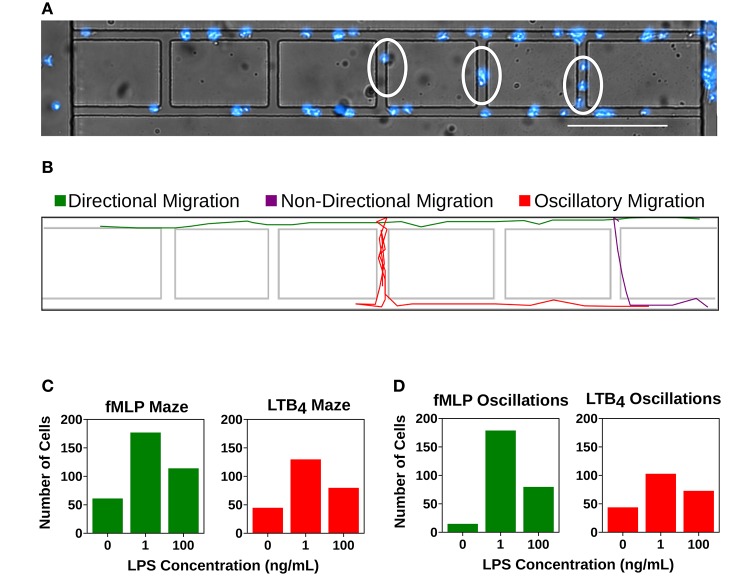
Super-low dose LPS primes neutrophils for dysfunctional chemotaxis. Quantification of cells entering the mazes within the microfluidic device or displaying an oscillatory migration pattern. **(A)** dHL-60 cells (blue) migrating within the microfluidic device. Cells within the linear migration channel are migrating directionally toward the chemoattractant gradient. Circled cells are displaying non-directional migration as they are migrating within the mazes. Scale Bar = 150 μm. **(B)** Computational recreation of cell migration tracks depicting directional migration (green), non-directional migration (purple), and oscillatory migration (red). **(C)** Number of “lost” cells that entered the maze while migrating toward fMLP (green) and LTB_4_ (red) in the unstimulated group (fMLP: n = 61, LTB_4_: 44), the group stimulated with super-low dose LPS (fMLP: n = 177, LTB_4_ = 129), and the group stimulated with high-dose LPS (fMLP: n = 114, LTB_4_: 79). Super- low dose LPS priming significantly increases the number of “lost” cells in both fMLP and LTB_4_ conditions compared to both control and high dose LPS treatment groups. **(D)** Number of cells that displayed oscillatory migration patterns while migrating toward fMLP (green) and LTB_4_ (red) in the unstimulated group (fMLP: *n* = 14, LTB_4_: 43), the group stimulated with super-low dose LPS (fMLP: *n* = 178, LTB_4_ = 102), and the group stimulated with high-dose LPS (fMLP: *n* = 79, LTB_4_: 72). Super-low dose LPS priming significantly increases the number of oscillatory cells in both fMLP and LTB_4_ conditions compared to both control and high dose LPS treatment groups. Data is representative of one experiment, however experiment was repeated at least 3 times.

We compared dHL-60 cells migration patterns after 0, 1, and 100 ng/ML LPS overnight treatment. Directional migration was defined as a cell that did not change direction in the x or y plane (Figures 5A,B and Supplementary Video 4). We measured the number of cells that displayed non-directional migration, in which cells become “lost” and are unable to follow the stronger chemoattractant gradient and therefore enter the maze ladder rung (Figures 5A,B and Supplementary Video 5). We also measured the number of cells that displayed oscillatory migration, in which the cell changed directions in the x or y plane at least three time (Figure 5B and Supplementary Video 6). We showed that super-low dose LPS treatment cause a higher number of cells to become display non-directional migration. The number of cells that entered the ladder rung in the LTB_4_ condition after super-low dose LPS treatment increased from 44 to 129 compared to the unprimed control (Figure 5C). A similar trend was also observed in the fMLP condition after super-low dose LPS treatment where cells entering the ladder increased from 61 to 178 (Figure 5C). Priming with high dose LPS increased cell non-directionality compared to the unprimed control cells (114 vs. 61 toward fMLP and 79 vs. 44 toward LTB_4_), but were only 75% as non-directional compared to the super-low dose treatment (114 vs. 177 toward fMLP and 79 vs. 129 toward LTB_4_) (Figure 5C). We also observed an increase in oscillatory migration with super-low dose treatment of dHL-60s (Figure 5D). The number of cells that displayed oscillatory migration toward fMLP increased from 14 to 178 compared to the unprimed control (Figure 5D). Similarly, the number of cells that displayed oscillatory migration toward LTB_4_ increased from 43 to 72 compared to the unprimed control (Figure 5D).

### Priming With LPS Increases Spontaneous Migration in dHL-60s

**Figure 6:**
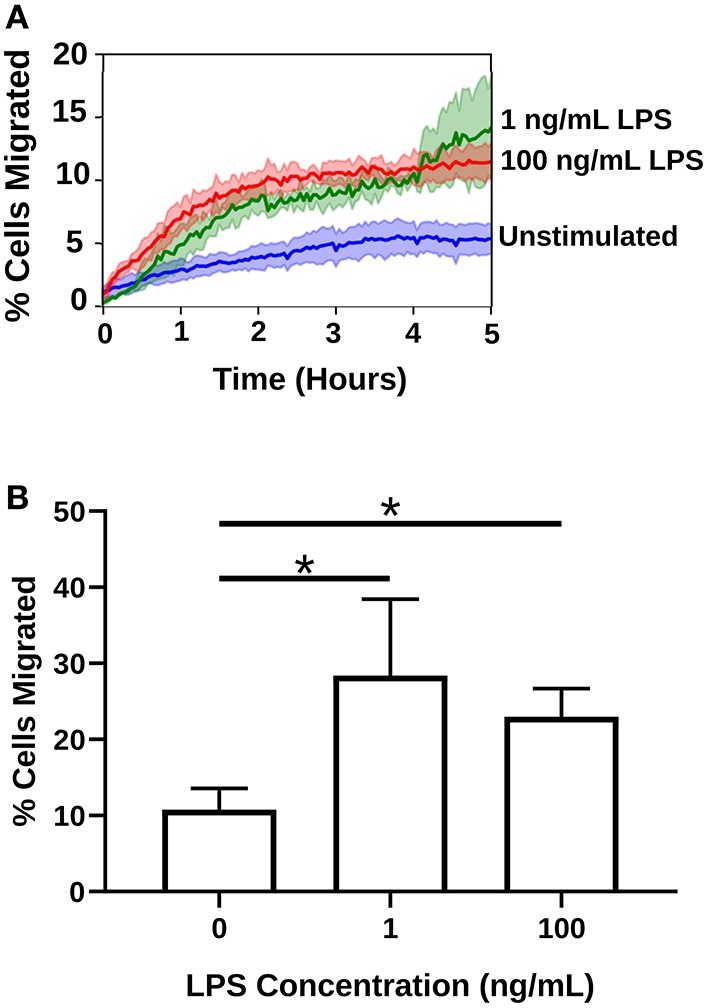
LPS priming increases spontaneous migration of neutrophils in the absence of chemoattractant. Migration counts of neutrophils (dHL-60s) that migrated in the absence of chemoattractant (*n* = 3 separate experiments). **(A)** High and super-low dose LPS treatment significantly increases the percentage of cells that spontaneously migrate into the migration channels of the microfluidic competitive chemotaxis-chip in the absence of chemoattractant. Blue depicts the unstimulated neutrophils, green the neutrophils stimulated with a super-low dose of LPS, and red the neutrophils stimulated with a high dose of LPS. **(B)** Percentages of migrating cells in the absence of chemoattractant in all three treatment groups. LPS stimulation significantly increases spontaneous dHL-60 cell migration in the absence of chemoattractant. Data expressed as means and standard deviations. *p < 0.05.

We have previously developed a microfluidic device that identified a sepsis-specific spontaneous migration signature displayed by isolated neutrophils originating from septic patients (1). In order to probe whether programming dHL-60 cells with LPS could recapitulate this spontaneous migration phenotype, we compared cell migration in the absence of chemoattractant with and without pre-treatment with LPS [1 and 100 ng/mL]. In unstimulated cells, only ~10% of dHL-60s migrated in the absence of chemoattractant. After priming with super-low dose LPS [1 ng/mL], spontaneous migration significantly increased to ~30% of cells (*p* = 0.0430) (Figure 6 and Supplementary Video 7). This finding illustrates that the “memory or training” of neutrophils considerably impact future migratory phenotypes.

## Discussion

In this study, we establish an effective microfluidic platform for the quantitative analysis of dHL-60 cells migration after LPS priming. For the first time, we demonstrate the significant effect that pre-treatment with super-low dose LPS has on neutrophil migratory decision-making. Super-low dose LPS pretreatment shifted dHL-60 cells to migrate preferentially toward an intermediary, inflammatory chemoattractant (LTB_4_) and increased spontaneous migration patterns. Furthermore, our results provide quantitative evidence, at the single-cell level, that neutrophil priming with varying levels of LPS influences subsequent migratory phenotype in opposing manners. Priming with high levels of LPS increases dHL-60 cells migration, whereas super-low dose LPS priming reverses the chemoattractant priorities of dHL-60 cells. Our data provide a range of quantitative characterizations of dHL-60 cell chemotaxis after LPS priming, including oscillatory migration patterns, directionality and cell velocity.

Although significant progress has been made in understanding the role of neutrophil activation in inflammation, dissecting the decision-making processes in different priming states is hampered by the complexity of *in vivo* conditions and the lack of detail of current *in vitro* assays. Microfluidics are emerging as an important tool for precisely quantifying neutrophil migratory phenotypes (1, 38–40). Compared to transwell (20), under-agarose, or Zigmond chamber assays, microfluidic systems provide extremely stable, linear gradients and allow direct observations and precise measurement of individual neutrophils during their migration. The microfluidic competitive chemotaxis-chip (μC^3^) presented in this study enables migratory decision-making of neutrophils to be observed simultaneously. The measurements enabled by the microfluidic device would not have been possible using traditional tools for observing cell migration. We are able to position a single neutrophil between two competitive signals and decipher how the neutrophil makes a decision which signal to follow. The intrinsic complexity of immune cell decision-making processes has been elusive for experimental immunologists despite expansive experimental studies with conventional reductionist cellular and molecular approaches. Engineering novel technologies to probe the competitive behavior of cells in precisely controlled environments is key to defining the diverse repertoires of cellular activation and differentiation states. A key feature of the assay is the ability to probe the effects of priming dHL-60 cells with varying levels of LPS. On the other hand, microfluidic cell analyses often requires specialized research facilities, such as microfabrication and live cell microscopy labs, as well as highly-skilled personnel to perform the experiments and analyze the data. We have adapted our design to be the first pump-free stand-alone microfluidic dual gradient device, which does not require external instrument controls, such as syringe pumps. The relatively simple and compact design of our microfluidic platform has the scaling potential to enable high-throughput screening of the priming effects of many different inflammatory mediators on a single chip by integrating multiple test units in parallel. In addition, we have developed an improved image analysis method to allow automated single cell tracking analysis, thus eliminate the need of lengthy and laborious post-experiment tracking analysis and permit instant result reporting.

This study confirms that the previous concept of LPS priming observed in monocytes and macrophages extends to neutrophil functional dynamics. Lower doses of LPS can induce a state of tolerance to subsequent toxic doses of LPS (37), but extremely low doses have an opposite effect, priming the immune system for an even more violent response to subsequent challenge. Microfluidic analysis of neutrophil chemotaxis has been recently demonstrated for successful diagnosis of sepsis (1, 4, 41). In sepsis, the immune response that is initiated by an invading pathogen fails to return to homeostasis, thus culminating in a pathological syndrome that is characterized paradoxically by sustained excessive inflammation and immune suppression (42). Correspondingly, pre-conditioning of experimental mice with super-low dose LPS exacerbate sepsis mortality (8). Our results show that pre-treating dHL-60 cells with super-low dose LPS can recapitulate many of the dysfunctional migration phenotypes observed in the septic, mouse model including elevated random migration and skewed migratory preference toward sterile inflammatory signals such as LTB_4_. Our data collected with an innovative, reductionist approach microfluidic platform using well-controlled dHL-60 cells pre-conditioned with super-low dose LPS are consistent with the previous animal study that reported increased neutrophil infiltration in multi-organs such as liver and spleen from septic mice pre-conditioned with super-low dose LPS *in vivo* (8). Recent studies further demonstrated that interruption or reversal of the impaired migration and antimicrobial function of neutrophils improves the outcome of sepsis in animal models (43). We also recently reported on oscillatory and spontaneous migration patterns in primary human peripheral blood neutrophils isolated from burn patients with sepsis (1). Spontaneous neutrophil migration is a unique phenotype, typical for patients with major burns during sepsis and often-observed one or two days before the diagnosis of sepsis is confirmed. The spontaneous neutrophil migration phenotype is rare in patients with major burns in the absence of sepsis, and is not encountered in healthy individuals. The recapitulation of this dysfunctional migratory phenotype in dHL-60 cells treated with super-low dose LPS is unprecedented and may shed light on the underlying pre-conditions that drive neutrophil dysfunction in sepsis. Further understanding the effects of super-low dose LPS on neutrophil function and decision-making will give insight into the effects of super-low level inflammation on future clinical outcomes. Furthermore, the device presented in this paper may be utilized to understand how programming neutrophils with pro-resolution mediators can restore the neutrophil migratory compass in inflammatory diseases, such as sepsis. One limitation of our study is that we fail to define the impact of neutrophil-neutrophil cross-talk. It is likely that dHL60 cells primed with LPS will produce an increase in pro-inflammatory mediators, including chemokines (LTB_4_) that may affect neighboring neutrophil migratory behaviors (8, 44–48). In the future, it will be possible to integrate biosensors in the microfluidic platform to measure neutrophil phenotypes beyond migration, including cytokine secretion levels. Measurements of single-cells migratory trajectories will also enable us to statistically determine if neutrophils are more likely to follow a similar path as a preceding neutrophil. The type of microfluidic platform described in this study will also enable us to measure migratory decision-making of heterogeneous populations of primed and unprimed neutrophils to answer the complex question of whether primed neutrophils will influence the migration of unprimed cells. Furthermore, we can investigate heterogenous populations of differing immune cells (e.g., neutrophils and TH17 cells or macrophages) (38, 49).

Advances in understanding of neutrophil behavior will come not only from molecular biology studies, but also from neutrophil phenotypic studies enabled by novel microfluidic platforms. In the future, we can engineer platforms with integrated biosensors (e.g., to quantify cytokine secretion) to measure other competitive behaviors of single immune cells, including differentiation or dynamic interaction with pathogens. In this study, we used a neutrophil-like differentiated human promyelocytic leukemia cell line (HL-60). In the future, we can use our platform to quantify neutrophil migratory decision-making from primary neutrophils isolated from mice models of sepsis or human septic patients. This study shows that super-low dose [1 ng/mL] LPS priming can significantly magnify spontaneous migration of neutrophils and redirect the neutrophil compass to favor pro-inflammatory chemoattractant signals. Further study of the effects of neutrophil priming or memory on migratory decision-making is warranted. A deeper understanding of neutrophil priming mechanisms may ultimately provide the basis for intervention strategies that would enable appropriate infiltration of phagocytes into inflammatory sites while minimizing neutrophil-mediated tissue injury.

## Materials and Methods

### Device Design and Fabrication

The microfluidic platform was designed with 2 opposing chemoattractant reservoirs and a central cell-loading channel (Figure 2A). The cell-loading chamber is connected to the chemoattractant reservoir by perpendicular cell migration ladders that enable precise measurements of cell directional migration and oscillatory migration. The master wafer was fabricated using standard photolithographic technologies with Mylar photomasks (FineLine Imaging, Colorado Springs, CO). Polydimethylsiloxane (PDMS) (Sylgard 184, Elsworth Adhesives, Wilmington, MA) microfluidic devices were made by replica molding from the master wafer. Briefly, PDMS and curing agent were combined at a 10:1 ratio, mixed thoroughly, and poured over the master wafer. PDMS was then degassed for 4 h and baked at 65°C overnight. The PDMS was then peeled from the master wafer, and channel inlets and outlets punched. The two outer chemoattractant loading chamber ports and central neutrophil loading ports were punched using a 1 mm puncher (Harris Uni-Core, Ted Pella Inc., Redding, CA). Each device was then cut out using an 8 mm puncher Following oxygen plasma treatment (Nordson March, Concord, CA), devices and 6-well glass-bottom plates (MatTek Corp. Ashland, MA) were bonded at 80°C on a hotplate for 10 min.

### Preparation of Microfluidic Migration Assay

To increase neutrophil adhesion to surface and to passivate device surface, 50 μL fibronectin (Sigma-Aldrich, St. Louis, MO) [11 μg/mL] was added to the top of the device. Fibronectin, a large glycoprotein, is one of the best-characterized cell adhesion-promoting extracellular matrix proteins (ECM) and is one of the most abundant proteins found in the human ECM (50). Fibronectin has been shown to increase the migration rate of neutrophils and has been used by us in previous microfluidic-based migration studies (1, 51). The device was then placed in a vacuum desiccator for 10 min and the fibronectin solution filled all of the channels as the air was displaced from the PDMS. The devices were then allowed to dry at room temperature for 30 min and the fibronectin absorbed to the glass and PDMS channel surfaces. The devices were then covered with complete media (4 mL). Chemoattractants were diluted using complete media. Ten microliters of each chemoattractant solution (N-Formylmethionine-leucyl-phenylalanine (fMLP, Sigma-Aldrich, St. Louis, MO) [10 nM] and Leukotriene B_4_ (LTB_4_, Cayman Chemical, Ann Arbor, MI) [100 nM] was then loaded into the chemoattractant reservoirs within the microfluidic device using a gel loading pipette tip. Optimal chemoattractant concentrations were chosen to induce maximal dHL-60 cell migration, are clinically relevant and match those previously reported (Supplementary Figure 1) (1, 38, 52–55). Tetramethylrhodamine fluorescent dextran (10,000 Da MW, Thermo Fisher Scientific, Waltham, MA) and Fluorescein fluorescent dextran (10,000 Da MW, Thermo Fisher Scientific, Waltham, MA) were added for visualization of the chemoattractant gradients in the device. dHL-60 cells [500,000 cell/10 μL] were loaded into the central cell-loading chamber within the microfluidic device using a gel loading pipette tips. The media surrounding the device was then removed and replaced with new complete media.

### Neutrophil Preparation and Treatments

Human promyelocytic leukemia cells (HL-60 CCL-240, American Type Culture Collection ATCC, Manassas, VA) were cultured in complete media containing Iscove's Modified Dulbecco's Medium (IMDM, ATCC, Manassas, VA) supplemented with 10% fetal bovine serum (FBS, ATCC, Manassas, VA) at 37°C in 5% CO_2_, according to ATCC instructions. HL-60 cells were differentiated to a neutrophil-like state by adding dimethyl sulfoxide (DMSO, Sigma-Aldrich, St. Louis, MO) (1.5% to 1.5 10^5^ cells mL^−1^) for 5 days (26, 56) (denoted as dHL-60 cells). On the fourth day of differentiation, cells were stimulated with lipopolysaccharide (LPS, *Escherichia coli* 0111:B4, Sigma-Aldrich, St. Louis, MO) to a concentration of 1 ng/mL for super-low dose stimulation or 100 ng/mL for high dose stimulation and incubated overnight at 37°C in 5% CO_2_. Cell viability was measured using a Trypan Blue exclusion test using an automated cell counter (Bio-Rad TC20™). Immediately prior to the migration experiment, dHL-60 cells were stained with Hoechst solution (Thermo Fisher Scientific, Waltham, MA) at a concentration of 20 mM for 10 min at 37°C in 5% CO_2_. Before migration assays, dHL60s were spun down (130G, no break) at RT for 7 min and washed with PBS to remove any dead cells. Viability of dHL-60 cells primed into the neutrophil loading zone of the microfluidic platform were >99% viable, as confirmed by Hoechst stain and neutrophil polarized morphology (change from round to elongated shape) upon live-cell attachment to fibronectin.

### Microscopy and Analysis

Time-lapse imaging experiments were performed at 37°C with 5% carbon dioxide on a fully-automated Nikon TiE microscope, using a Plan Fluor 10x Ph1 DLL (NA = 0.3) lens. Image capture was performed using NIS-elements (Nikon Inc., Melville, NY) and analysis performed using the ImageJ (TrackMate) (57). Images were recorded using fluorescent and bright-field channels at two and a half minute intervals for 5 h. dHL-60 cell migration was quantified as followed: (1) percentage of cells migrating fully toward chemoattractant reservoirs, (2) velocity of migration, and (3) directionality of migration. Cell counts were conducted using ImageJ software (NIH). Cell tracking was performed automatically from DAPI images for the time-lapse sequences. All custom tracking and analysis algorithms are available for download at (https://github.com/boribong/Single-Cell-Migration-Tracking). Cell motility definitions are detailed in Table 1.

### Statistical Analysis

All experiments were performed and replicated at least 3 times, unless otherwise stated. Statistical analysis was performed using Prism software (GraphPad Software, La Jolla, CA). Data expressed as means ± standard deviations. To compare the parameters of dHL-60 cells migration between unstimulated, 1 ng/mL and 100 ng/mL overnight LPS treatment, we used a Student's *t*-test and differences were considered statistically significant for *p* < 0.05.

## Author Contributions

CJ and LL conceived the experiment(s). BB and CJ conducted the experiments and analyzed data. ML contributed to microfluidic device design. CJ, BB, and LL wrote the manuscript.

### Conflict of Interest Statement

The authors declare that the research was conducted in the absence of any commercial or financial relationships that could be construed as a potential conflict of interest.
