# Supplementary material for: Super-Low Dose Lipopolysaccharide Dysregulates Neutrophil Migratory Decision-Making
Source: Front Immunol. 2019 Mar 12;10:359. doi: 10.3389/fimmu.2019.00359 (PMC6422936; doi:10.3389/fimmu.2019.00359)
Supplement: Supplementary file 8 [file Data_Sheet_1.docx]

Supplementary Material

Super low-dose lipopolysaccharide dysregulates neutrophil migratory decision-making

Brittany P. Boribong, Mark J. Lenzi, Liwu Li*, Caroline N. Jones*

*** Correspondence:** Corresponding Authors: CNJ (email: [jonescn@vt.edu](mailto:jonescn@vt.edu)) and LL ([lwli@vt.edu](mailto:lwli@vt.edu))

Supplementary Figure 1. **Dose-response of dHL-60 cell migration toward fMLP and LTB_4_ chemoattractants.** Neutrophil (dHL-60 cells) migration counts toward 1, 10, 50, 100nM and 1μM concentrations of **(A)** fMLP and **(B)** LTB4 in the migration channels. n = 1. **(A)** Unstimulated dHL-60 cells preferentially migrate toward 10nM fmLP. **(B)** Unstimulated dHL-60 cells preferentially migrate toward 100nM LTB_4_.

Supplementary Figure 2. **Effects of high dose and super-low dose LPS on cell viability.** dHL-60 cell viability is increased by 2% when stimulated with super-low dose LPS in comparison to the unstimulated control whereas stimulation with high dose LPS has no impact.

Supplementary Video 1. **Unstimulated dHL-60 cells migrating toward fMLP and LTB_4_ within the μC^3^.** dHL-60 cells (blue) preferentially migrating toward fMLP (green) over LTB_4_ (red) within the μC^3^. Scale Bar = 500μm.

Supplementary Video 2. **dHL-60 cells stimulated with super-low dose LPS migrating toward fMLP and LTB_4_ within the μC^3^.** dHL-60 cells (blue) stimulated with super low-dose LPS [1 ng/mL LPS] preferentially migrating toward LTB_4_ over fMLP (green) within the μC^3^. Scale Bar = 500μm.

Supplementary Video 3. **dHL-60 cells stimulated with high dose LPS migrating toward fMLP and LTB_4_ within the μC^3^.** dHL-60 cells (blue) stimulated with high-dose LPS [100 ng/mL LPS] preferentially migrating toward fMLP (green) over LTB_4_ (red) within the μC^3^. Scale Bar = 500μm.

Supplementary Video 4. **Computational simulation of a dHL-60 cell migrating directionally within the μC^3^.**

Supplementary Video 5. **Computational simulation of a dHL-60 cell migrating non-directionally within the μC^3^.**

Supplementary Video 6. **Computational simulation of a dHL-60 cell displaying oscillatory migration within the μC^3^.**

Supplementary Video 7. **dHL-60 cells stimulated with super-low dose LPS migrating in the absence of chemoattractant within the μC^3^.** dHL-60 cells (blue) stimulated with super low-dose LPS [1 ng/mL LPS] displaying spontaneous migration in the absence of chemoattractant within the μC^3^. Scale Bar = 500μm.
