# Supplementary figures and images for: Super-Low Dose Lipopolysaccharide Dysregulates Neutrophil Migratory Decision-Making
Source: Front Immunol. 2019 Mar 12;10:359. doi: 10.3389/fimmu.2019.00359 (PMC6422936; doi:10.3389/fimmu.2019.00359)

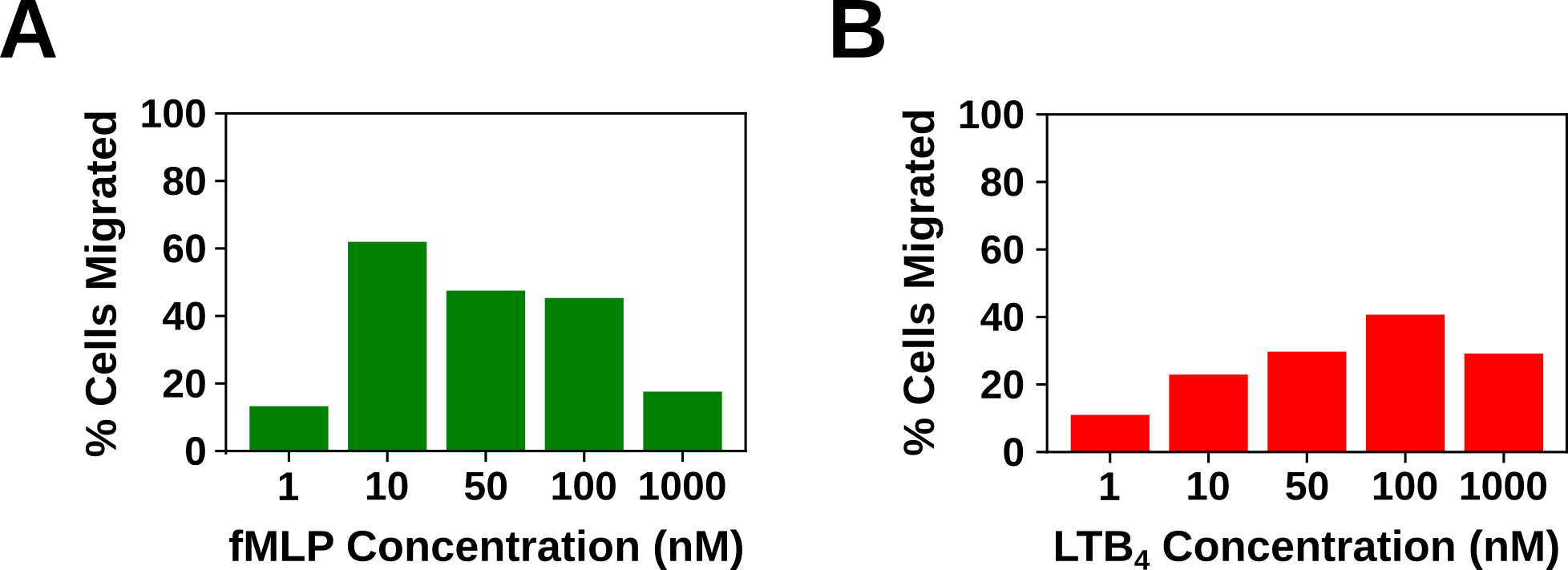

Supplement: Supplementary file 9 [file Image_1.TIFF]

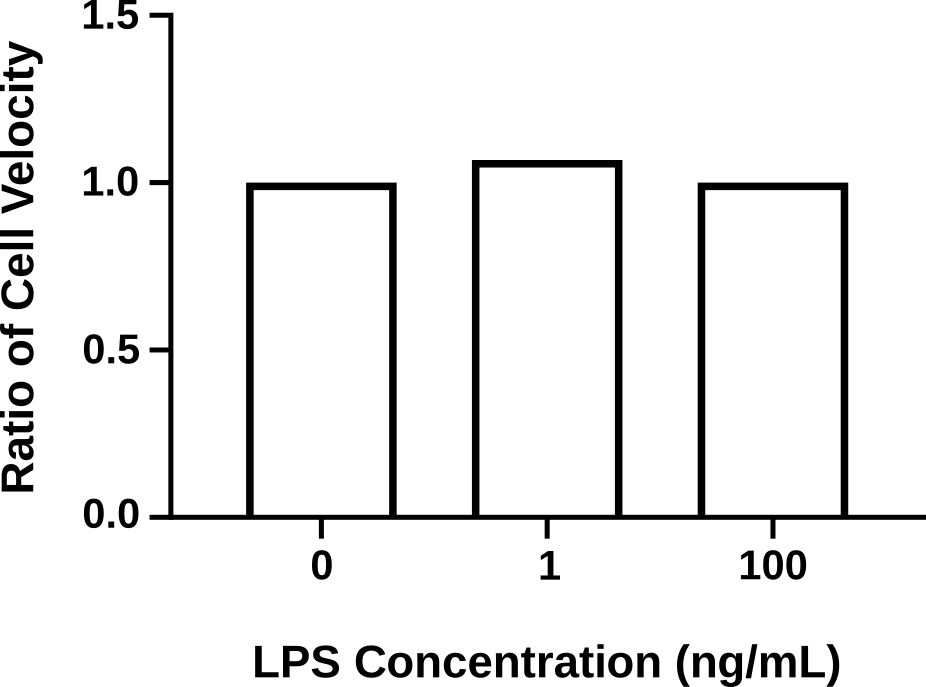

Supplement: Supplementary file 10 [file Image_2.TIFF]
